# Long-term effects of aromatase inhibitor withdrawal on bone mineral density in early breast cancer patients: 10-year follow-up results of the BREX study

**DOI:** 10.1007/s10549-024-07252-7

**Published:** 2024-04-01

**Authors:** Carl Blomqvist, Leena Vehmanen, Pirkko-Liisa Kellokumpu-Lehtinen, Riikka Huovinen, Johanna Ruohola, Heidi Penttinen, Harri Sievänen, Riku Nikander, Meri Utriainen, Tiina Saarto

**Affiliations:** 1 Department of Oncology, Helsinki University Central Hospital, Comprehensive Cancer Center and University of Helsinki, Helsinki, Finland; 2 Department of Oncology, Faculty of Medicine and Health Technology, Tampere University Hospital, University of Tampere, Tampere, Finland; 3 Department of Oncology and Radiotherapy, Turku University Hospital, Turku, Finland; 4 Cancer Society of Pirkanmaa, Tampere, Finland; 5 The UKK Institute for Health Promotion Research, Tampere, Finland; 6 Faculty of Sport and Health Sciences, University of Jyväskylä, Jyväskylä, Finland

**Keywords:** Aromatase inhibitor withdrawal, Bone loss, Breast cancer

## Abstract

**Purpose:**

We aimed to provide long-term bone mineral density (BMD) data on early breast cancer patients of the BREX (Breast Cancer and Exercise) study. The effects of exercise and adjuvant endocrine treatment 10 years after randomization were analyzed, with special emphasis on aromatase inhibitor (AI) therapy discontinuation at 5 years.

**Methods:**

The BREX study randomized 573 pre- and postmenopausal breast cancer patients into a 1-year supervised exercise program or a control group. 372 patients were included into the current follow-up analysis. BMD (g/cm^2^) was measured by dual-energy X-ray absorptiometry at lumbar spine (LS), left femoral neck (FN), and the total hip. Separate groups were displayed according to baseline menopausal status, and whether the patient had discontinued AI therapy at 5 years or not.

**Results:**

The BMD change from 5 to 10 years did not significantly differ between the two randomized arms. AI discontinuation at 5 years had statistically significant BMD effects. The FN BMD continued to decrease in patients who discontinued AI therapy during the first 5-year off-treatment, but the decrease was three-fold less than in patients without AI withdrawal (− 1.4% v. − 3.8%). The LS BMD increased (+ 2.6%) in patients with AI withdrawal during the first 5 years following treatment discontinuation, while a BMD decrease (-1.3%) was seen in patients without AI withdrawal.

**Conclusion:**

This study is to our knowledge the first to quantify the long-term impact of AI withdrawal on BMD. Bone loss associated with AI therapy seems partially reversible after stopping treatment. Trial registration: http://www.clinicaltrials.gov/ (Identifier Number NCT00639210).

**Supplementary Information:**

The online version contains supplementary material available at 10.1007/s10549-024-07252-7.

## Introduction

Breast cancer survivors are at an increased risk for osteoporosis and fracture compared with women in general [1]. Many adjuvant breast cancer therapies interfere with bone turnover and predispose patients to treatment-related bone loss [2].

Endocrine therapy with aromatase inhibitors (AIs) letrozole, anastrozole, and exemestane inhibits peripheral estrogen production. Estrogen deficiency leads to accelerated bone loss and increases the risk of fracture, especially in postmenopausal women [3, 4]. AI therapy causes a two- to four-fold increase in bone loss compared with physiologic postmenopausal loss of bone mineral density (BMD) [4]. Tamoxifen has partial estrogen-agonist and bone-sparing effects among postmenopausal, albeit not premenopausal, women [2, 4–6].

Anastrozole and tamoxifen were compared as breast cancer adjuvant treatment for postmenopausal women in the ATAC trial. In the bone sub-study of ATAC, the median BMD decreased by − 6.1% at the lumbar spine (LS) and − 7.2% at the total hip during 5 years of anastrozole therapy, while BMD increased in tamoxifen users (LS + 2.8% and total hip + 0.7%) [5]. 2 years after completion of the AI therapy, however, a partial recovery in the LS BMD and a stabilization at the total hip were noticed [7].

In the IES breast cancer study, postmenopausal patients were randomized after 2–3 years of adjuvant tamoxifen to continue tamoxifen or switch to exemestane to complete a total of 5 years of endocrine therapy. The BMD decline seen during exemestane therapy was partially reversed in the LS and stabilized at the hip 2 years after treatment withdrawal [8]. The IBIS-II trial compared anastrozole with placebo in postmenopausal women at high risk of developing breast cancer. Like in the adjuvant trials, the negative effects of AI on BMD were partially reversed 2 years after stopping treatment [9].

Bone loss associated with AIs thus seems to be at least partially reversible after treatment discontinuation, particularly at the LS. However, the follow-up periods after AI withdrawal of the adjuvant ATAC and IES trials as well as of the IBIS-II breast cancer prevention study were short, only 2 years. No long-term data on BMD recovery has been available so far [7–9].

The primary objective of the BREX (Breast Cancer and Exercise) study was to investigate the effects of physical exercise on BMD and quality of life in breast cancer survivors. The 12-month exercise program prevented hip bone loss in premenopausal patients for 3 years, while no training effects on BMD were seen in postmenopausal women [10, 11]. The current analysis aimed to study the long-term BMD outcomes with special emphasis on effects of endocrine therapy withdrawal 5 years after treatment discontinuation.

## Patients and methods

The BREX (Breast Cancer and Exercise) study was an open, randomized controlled exercise intervention trial. A total of 573 pre- and postmenopausal women aged from 35 to 68 years with newly diagnosed breast cancer were enrolled into the study between September 2005 and September 2007 from the Departments of Oncology in Helsinki, Tampere, and Turku University Hospitals, Finland.

Details of the exercise intervention and the study protocol have been presented in our previous publication [10]. Inclusion and exclusion criteria for the BREX study are provided as Supplemental material. Patients were randomly allocated into 1-year exercise training or control groups. Randomization was centralized and stratified for study centers, menopausal status, endocrine treatment in postmenopausal women (aromatase inhibitor vs. tamoxifen vs. no endocrine treatment), and for age in premenopausal women (below or above 45 years).

The local ethical committee of Helsinki University Hospital approved the study protocol. Informed consent was obtained from all individual participants included in the study. The trial was registered in the Helsinki and Uusimaa Hospital District Clinical Trials Register (www.hus.fi; 210,590) and http://www.clinicaltrials.gov/ (Identifier Number NCT00639210).

All patients were treated with adjuvant chemotherapy and/ or endocrine therapy. Radiotherapy was given after breast-conserving surgery. Radiation to the chest wall after mastectomy and regional nodal irradiation was given according to local clinical guidelines. Chemotherapy and radiotherapy had to be completed and endocrine treatment started no later than 4 months before study enrollment.

Three different chemotherapy regimens at 3-week intervals were used: six cycles of FEC (5-fluorouracil, epirubicin, and cyclophosphamide), three cycles of docetaxel followed by three cycles of FEC, or three cycles of docetaxel followed by three cycles of EC (epirubicin and cyclophosphamide), the last regimen combined with capecitabine for 2 weeks as per the FinXX protocol [12]. The recommended endocrine treatment was tamoxifen for premenopausal patients and aromatase inhibitor, tamoxifen, or both sequentially for postmenopausal patients.

The total duration of the exercise training program was 12 months, and it comprised supervised exercise and home training. The exercise group attended either one 60-min supervised session of step aerobics or circuit training on alternate weeks and three home training sessions per week. The training was progressive. A detailed description of the exercise sessions has been published previously [10]. Patients in the control group were recommended to maintain their habitual level of physical activity and exercise.

BMD was measured at baseline, after completion of the 12-month intervention and at 3, 5 and 10 years thereafter. Body height and weight were measured using standard methods. BMD (g/cm^2^) was measured by dual-energy X-ray absorptiometry (DXA) at LS (vertebrae L1–L4), left femoral neck (FN), and the total hip. In Helsinki, BMD was measured with Hologic Discovery A and in Tampere, with Lunar Prodigy Advance, at baseline and during the follow-up measurements. In Turku, Hologic QDR-4500 was used at baseline and either Hologic QDR-4500, Medilink Osteocore III, or Medilink Medix during the follow-up. Since different DXA brands were employed and their absolute BMD values are not directly comparable, the relative changes in BMD data were compared in the statistical analyses.

**Fig. 1:**
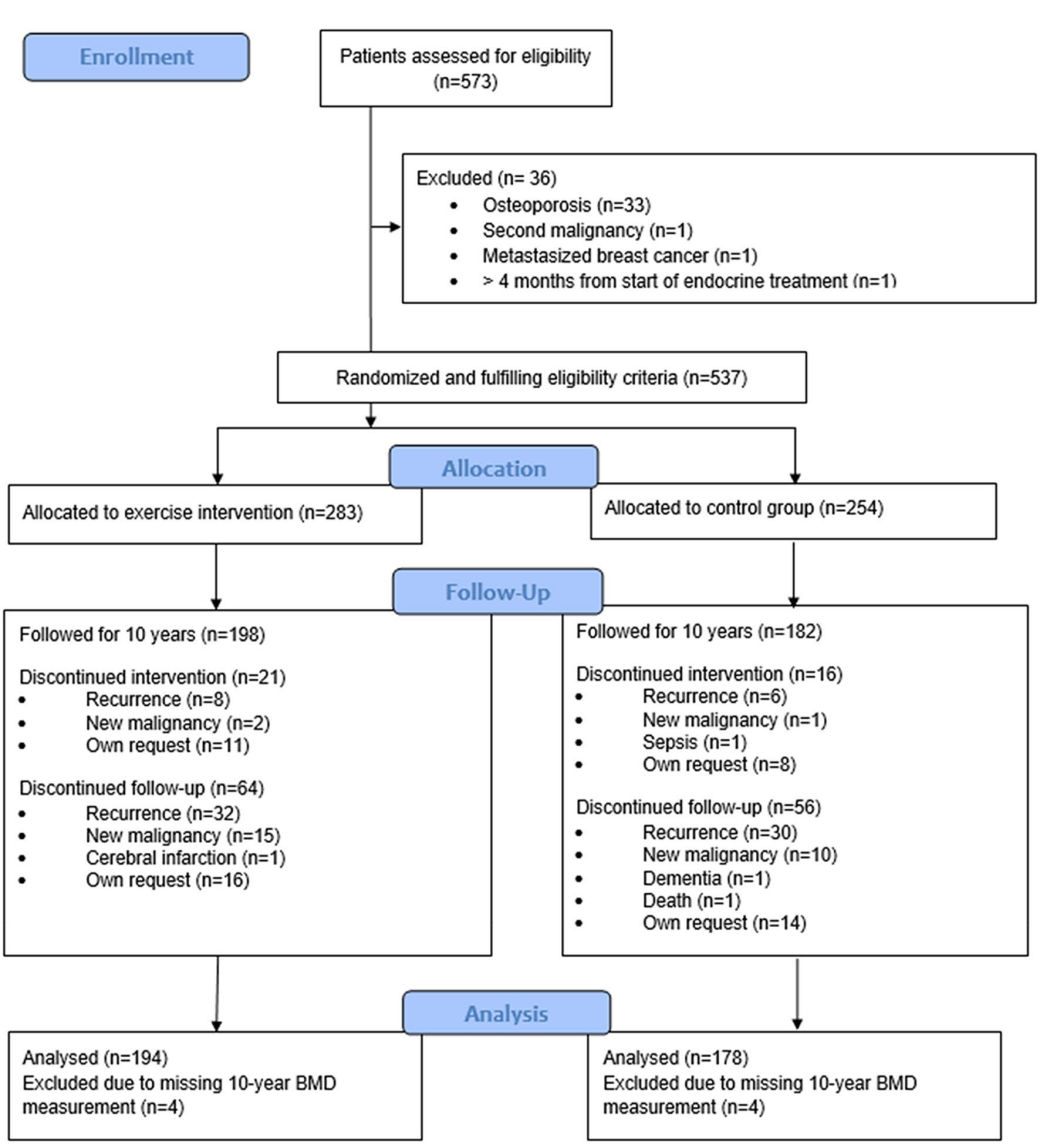
CONSORT diagram of the Breast Cancer and Exercise (BREX) study

A total of 573 patients were randomized into exercise and control groups, of whom 537 received the allocated intervention (284 in the exercise and 253 in the control group). A flow-chart of patient inclusion and reasons for exclusion from the present study is shown in Fig. 1. Altogether, 372 patients attended the 10-year follow-up visit, had BMD measurements at the 10-year follow-up, and were included in the current analysis. Of these women, 167 were premenopausal and 205 postmenopausal.

The division into pre- and postmenopausal groups was based on the baseline menstrual status of the patients. The definition of postmenopausal status was at least 12 months of amenorrhea and for those, whose menstruation status could not be assessed due to hysterectomy or hormonal intrauterine device (IUD), patients < 50 years were classified as premenopausal, patients ≥ 55 years as postmenopausal, and those in between according to whether the FSH level was in the postmenopausal range (> 30 IU/l) or not. Most of the premenopausal patients went through menopause during the 10-year follow-up period of the study.

## Statistical methods

Patient characteristics were shown as proportions, means, or medians with standard deviation (SD) or range as appropriate for the characteristics of variable distribution. According to the study protocol separate analyses were done for patients who were pre- and postmenopausal at diagnosis. Endocrine treatment at baseline and after 5 years of follow-up was recorded. Separate groups were displayed according to menopausal status at baseline and whether the patient had discontinued AI therapy at 5 years or not. Adjuvant endocrine treatments for the whole 5-year period were summarized into five groups: none, discontinued, tamoxifen, AIs, or mixed. Patients using tamoxifen or a combination of tamoxifen plus LHRH-agonist were combined, as well as patients with AIs with or without LHRH-agonist therapy. The treatment was classified as discontinued if stopped within 4 years from baseline. The mixed category contained patients with sequential tamoxifen and AI therapy irrespective of sequence and one patient with tamoxifen followed by LHRH-agonist therapy.

Patients with AI therapy at 5 years and discontinuing this therapy within 2 years from the 5-year control were included in the AI discontinuing group. Patients with tamoxifen treatment, LHRH inhibitor therapy or no endocrine treatment at 5 years, and patients continuing tamoxifen or AI therapy beyond 5 years for at least 3 years were combined into the group not discontinuing AI therapy.

The effect of random allocation into exercise and control groups on BMD at five and 10 years was tested with the independent samples *t* test. Univariable analyses on the effect of baseline menopausal status and AI therapy discontinuation on BMD change from five to 10 years in the FN and LS were done with the independent samples t test. Multivariable analyses on the effect of these factors were done in a two-factor general linear model. 12 patients had used medication for osteoporosis on some occasion between the 5- and 10-year follow-up visits. A separate general linear model analysis was done excluding these patients as a sensitivity analysis. All analyses were done with SPSS version 28 and the threshold for significance was set at 0.05 for the effect of menopausal status and AI therapy on BMD, while Bonferroni correction for multiple comparisons was applied for testing the effect of random allocation of the exercise intervention.

## Results

372 patients attended the 10-year follow-up visit and had BMD measurements at the 10-year follow-up visit (Fig. 1). Patient characteristics at baseline and 5 years of follow-up are shown in Table 1. 309 women (83%) received adjuvant endocrine therapy at baseline. 16 patients (4%) discontinued endocrine therapy prematurely before the 5-year follow-up, while 293 (79%) continued endocrine therapy for at least 5 years. 265 patients (71%) discontinued their adjuvant endocrine therapy at 5 years; 96 patients (26%) discontinued tamoxifen, 1 patient LHRH therapy, and 168 patients (45%) AI therapy. Four patients (1%) continued tamoxifen and two (0.5%) AI therapy at 5 years and 22 patients (6%) switched tamoxifen treatment into an AI.

**Table 1:** Patient characteristics

Characteristics	Premenopausal		Postmenopausal	
	AI discontinued		AI discontinued	
	Yes (*n* = 28)	No (*n* = 139)	Yes (*n* = 140)	No (*n* = 65)
Age at baseline, years* (range)	48.8 (37–54)	46.4 (35–57)	58.5 (47–68)	58.3 (48–67)
T Ø, mm** (range), *n* = 371	18 (6–70)	18 (4–72)	20 (4–130)	16 (4–54)
N metastatic, ** (range)	1.5 (0–11)	1 (0–10)	1 (0–13)	1 (0–13)
ER + , *n*	28 (100%)	105 (76%)	140 (100%)	34 (52%)
Baseline				
BMI, kg/m^2^* (SD)	25.1 (3.2)	24.8 (4.0)	26.0 (4.0)	26.6 (4.1)
FN BMD, g/cm^2^ (SD), *n* = 371	0.79 (0.10)	0.81 (0.11)	0.78 (0.11)	0.79 (0.13)
LS BMD, g/cm^2^ (SD), *n* = 371	1.00 (0.13)	1.02 (0.13)	0.99 (0.13)	0.99 (0.14)
5-year follow-up				
BMI, kg/m^2^* (SD), *n* = 364	26.4 (3.7)	26.0 (4.7)	26.6 (4.5)	26.6 (3.9)
FN BMD, g/cm^2^ (SD), *n* = 367	0.74 (0.10)	0.79 (0.11)	0.74 (0.10)	0.77 (0.12)
LS BMD, g/cm^2^ (SD), *n* = 368	0.93 (0.14)	0.97 (0.13)	0.94 (0.12)	0.98 (0.16)
Initial ET, *n* (%)				
Tamoxifen	23 (82)	109 (78)	40 (29)	17 (26)
Anastrozole	2 (7)^##^	0	96 (69)	13 (20)
Letrozole	1 (4)	0	3 (2)	2 (3)
Exemestane	1 (4)	0	1 (1)	0
Other	0	0	0	0
None	0	30 (22)	0	33 (51)
5-year ET summary, *n* (%)				
None	0	30 (22)	0	33 (51)
Discontinued^€€^	0	9 (7)	0	7 (11)
Tamoxifen^€€^	0	96 (69)	0	12 (19)
Aromatase inhibitor^€€^	5 (18)	0	99 (71)	1 (2)
Mixed^€€^	23 (82)	4 (3)	41 (29)	12 (19)
ET at 5 year, *n* (%)				
Tamoxifen	0	98 (71)^##^	0	24 (37)
Anastrozole	16 (57)^#^	1 (1)	105 (75)	1 (2)
Letrozole	6 (21)^#^	0	16 (11)	0
Exemestane	6 (21)	0	19 (14)	0
Other	0	1 (1)^€^	0	0
None	0	39 (28)	0	40 (62)

*n* number, *T* tumor size, *N* lymph nodes, *ER* estrogen receptor, *SD* standard deviation, *BMI* body mass index, *BMD* bone mineral density

*Mean, **median, ^#^two patients with LHRH-agonist, ^##^one patient with LHRH-agonist, ^€^LHRH-agonist, €€ see Statistical methods for explanation

The mean BMD was 0.765 (SD 0.11) and 0.743 g/cm^2^ (SD 0.11) in FN and 0.960 (SD 0.13) and 0.963 g/cm^2^ (SD 0.15) at LS at 5 and 10 years of follow-up, respectively. There were no statistically significant differences between the two randomized arms in the mean BMD values at 5 and 10 years of follow-up nor in change from 5 to 10 years (Table 2). For subsequent analyses, the two randomized arms were combined.

**Table 2:** Differences in BMD in the exercise and control groups at 5 and 10 years of follow-up

	Exercise (*n* = 194) BMD g/cm^2^, mean (SD)	Control (*n* = 178) BMD g/cm^2^, mean (SD)	*p*-value
5-year FN* (*n* = 367)	0.76 (0.11)	0.78 (0.12)	
Premenopausal	0.76 (0.10)	0.80 (0.12)	
Postmenopausal	0.75 (0.11)	0.75 (0.11)	
5-year LS* (*n* = 368)	0.95 (0.13)	0.97 (0.14)	
Premenopausal	0.94 (0.11)	0.99 (0.15)	
Postmenopausal	0.96 (0.14)	0.95 (0.13)	
10-year FN* (*n* = 370)	0.74 (0.11)	0.75 (0.12)	
Premenopausal	0.73 (0.10)	0.78 (0.12)	
Postmenopausal	0.74 (0.12)	0.73 (0.11)	
10-year LS* (*n* = 372)	0.97 (0.15)	0.96(0.15)	
Premenopausal	0.92 (0.13)	0.97 (0.16)	
Postmenopausal	0.99 (0.15)	0.97 (0.15)	
FN change from 5 to 10 years, % (*n* = 366)^#^	− 2.8 (6.0)	− 2.7 (5.0)	0.94
Premenopausal	− 3.7 (5.5)	− 3.2 (5.1)	0.49
Postmenopausal	− 2.0 (6.2)	− 2.4 (4.9)	0.67
LS change from 5 to 10 years, % (*n* = 368)^#^	+ 0.5 (6.6)	+ 0.4 (8.3)	0.92
Premenopausal	− 1.7 (7.0)	− 1.4 (9.5)	0.81
Postmenopausal	+ 2.2 (5.8)	+ 1.9 (6.8)	0.74

^#^Bonferroni-corrected significance threshold *p* = 0.008

### Changes of BMD according to menopausal and endocrine treatment status at 5 years

Mean FN BMD at 5 years was 0.784 g/cm^2^ (SD 0.11) in premenopausal and 0.750 g/cm^2^ (SD 0.11) in postmenopausal women. Mean FN BMD at 5 years was 0.742 g/cm^2^ (SD 0.10) in patients discontinuing AI therapy and 0.784 g/cm^2^ (SD 0.10) in the others (patients receiving tamoxifen, LHRH-agonist, no endocrine treatment, or continuing AI therapy).

FN BMD decreased by − 3.4% from 5 to 10 years in premenopausal patients and by − 2.2% in postmenopausal women (*p* = 0.03). FN BMD decreased by − 1.4% from 5 to 10 years in patients discontinuing AI at 5 years and by − 3.8% in the others (*p* = 0.00003).

Mean LS BMD at 5 years was 0.966 g/cm^2^ (SD 0.13) in premenopausal and 0.955 g/cm^2^ (SD 0.14) in postmenopausal women. Mean LS BMD was 0.941 g/cm^2^ (SD 0.12) in patients discontinuing AI therapy at 5 years and 0.976 g/cm^2^ (SD 0.14) in the others.

LS BMD decreased by − 1.5% from 5 to 10 years in premenopausal patients, while it increased by + 2.1% in postmenopausal women (*p* = 0.000003). LS BMD increased by + 2.6% from 5 to 10 years in patients discontinuing AI at 5 years, while it decreased by − 1.3% in the others (p = 0.0000007).

**Fig. 2:**
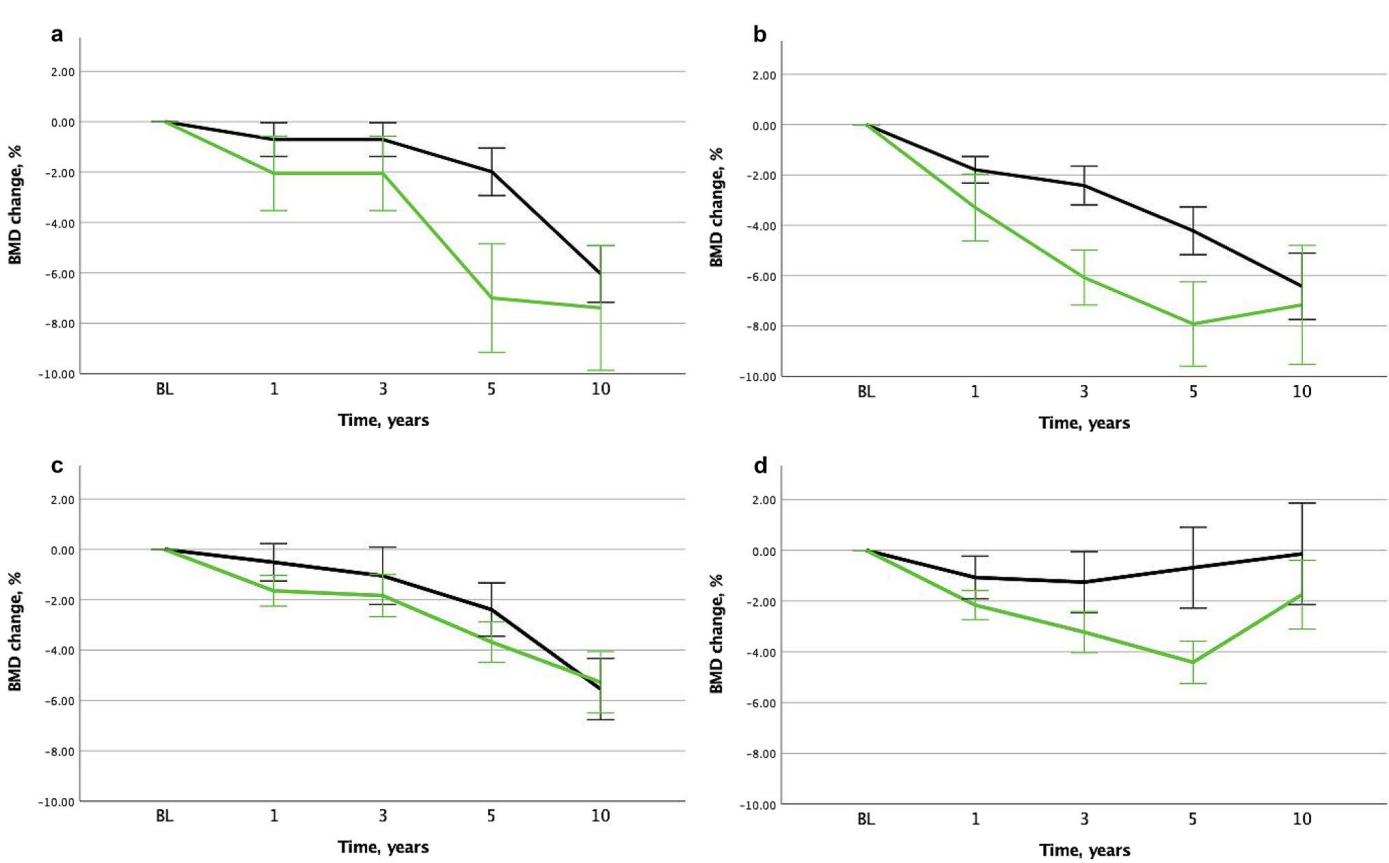
**a** Femoral neck BMD in premenopausal patients according to AI therapy discontinuation at 5 years. **b** Lumbar spine BMD s in premenopausal patients according to AI therapy discontinuation at 5 years. **c** Femoral neck BMD in postmenopausal patients according to AI therapy discontinuation at 5 years. **d** Lumbar spine BMD in postmenopausal patients according to AI therapy discontinuation at 5 years. *BL* baseline. Green line: AI discontinued at 5 years. Black line: Tamoxifen, LHRH-agonist treatment, no adjuvant endocrine therapy or AI continuing beyond 5 years

The BMD changes from 5 to 10 years of follow-up in patients discontinuing AI vs. not according to menopausal status are presented in Table 3 and BMD changes from baseline in Fig. 2.

**Table 3:** BMD changes from 5 to 10 years of follow-up in patients discontinuing AI vs. not according to menopausal status

	BMD change from 5 to 10 years, %						P^1^	P^2^	P^3^
	Premenopausal			Postmenopausal					
		AI discontinued after 5 years			AI discontinued after 5 years				
	All	Yes	No	All	Yes	No			
FN*, *n* = 366	− 3.4% (5.3)	− 0.1% (4.6)	− 4.1% (5.2)	− 2.2% (5.6)	− 1.7% (6.2)	− 3.2% (3.9)	0.63	0.0001	0.08
LS*, *n* = 368	− 1.5% (8.3)	+ 1.5% (4.8)	− 2.1% (8.7)	+ 2.1% (6.3)	+ 2.8% (6.8)	+ 0.6% (4.9)	0.03	0.002	0.47

P^1^ Menopausal status

P^2^ AI discontinuation

P^3^ Interaction between menopausal status and AI discontinuation

*Mean (SD)

A two-way general linear model testing BMD changes from 5 to 10 years according to pretreatment menopausal status and discontinuation of AI therapy is shown in Table 3. Discontinuation of AI therapy was highly significantly associated to BMD change in both FN (*p* = 0.0001) and LS (*p* = 0.002). The p-values for interaction between menopausal status and AI discontinuation were not significant, indicating that the effect of AI was similar in the menopausal groups. Menopausal status was significantly associated to LS BMD change (*p* = 0.03), while the effect of menopausal status on FN BMD failed to reach significance. The effect of AI discontinuation on FN (*p* = 0.0001) and LS BMD (*p* = 0.0005) remained highly significant after exclusion of patients on treatment for osteoporosis between 5 and 10 years (*n* = 355 and *n* = 357, respectively).

## Discussion

The aim of this follow-up analysis was to provide long-term BMD data of early breast cancer patients included in the BREX exercise trial, with special emphasis on AI therapy discontinuation. We have previously reported that the 1-year exercise intervention diminished BMD loss at the hip for 3 years, but by the fifth follow-up year no significant differences remained between the randomized groups [11]. The extended follow-up of the study patients and their systematic BMD measurements provided an opportunity of evaluating the long-term effects of adjuvant endocrine treatment on BMD.

Femoral neck BMD continued to decrease after AI withdrawal during 5-year off-treatment, but the decrease was three-fold less than in patients without AI withdrawal. AI withdrawal was associated with an increase in lumbar spine BMD during the first 5 years following treatment discontinuation. These results add to evidence from previous studies with shorter follow-up suggesting a decrease in hip bone loss and a partial BMD recovery at the LS after AI withdrawal.

Current treatment guidelines recommend considering adjuvant AI therapy for most postmenopausal women with early estrogen receptor (ER)-positive breast cancer and for high-risk premenopausal patients together with ovarian suppression [13, 14] AI therapy increases bone loss and the risk of fracture compared to estrogen receptor antagonists. Bisphosphonates and denosumab prevent bone loss associated with adjuvant AI therapy and bisphosphonates seem to even decrease the risk of cancer recurrence and death among women with postmenopausal breast cancer [2, 4, 15]. However, the optimal target population and regimen of the bone-modifying agents is currently not known.

According to a large meta-analysis of postmenopausal women with early breast cancer, the risk of fracture was 8.2% during 5 years of AI treatment [6]. In a real-world setting, the fracture incidence with 5 years of AI therapy has been reported to be as high as 20% [4]. After completion of AI treatment, the rate of fragility fractures decreases [4, 7, 8]. Less data is available on fracture risk among premenopausal women with early breast cancer. In a recent meta-analysis, the fracture risk was 6.4% in premenopausal women receiving ovarian suppression with AIs as compared to 5.1% in patients with ovarian suppression and tamoxifen [16].

In the IBIS-2 breast cancer prevention study, a significant BMD loss was seen during 5 years of anastrozole in postmenopausal women with normal BMD at baseline [17]. However, a mean increase of + 1.3% was seen in LS BMD during the first 2-year off-treatment while no significant change was observed in the hip BMD [9]. Following 5 years of anastrozole adjuvant therapy in the ATAC trial, the LS BMD increased by + 4.0% during the first 2-year off-treatment, while no significant changes were noted at the total hip [7].

The AI-associated BMD loss thus begins to resolve after treatment discontinuation, but the recovery seems to be only partial. In the current study, BMD was measured once at the end of the 5-year off-treatment period. Therefore, we cannot know whether and when a plateau in the BMD recovery was reached. Postmenopausal estrogen replacement therapy increases BMD especially during the first 2 years of treatment after which a plateau is achieved [18].

Main strengths of the current study are the long follow-up time, systematic BMD measurements, and a relatively large number of included patients. To our knowledge, the BREX study has the longest BMD follow-up reported after AI withdrawal. Endocrine therapy of the study patients was not randomized. The diversity of endocrine therapy regimens used reflects the real-world scenario, where patients often switch from one endocrine therapy to another due to, e.g., toxicity. Of note, only few of the study patients discontinued endocrine therapy prematurely, unlike in many previous reports [19]. Limitations of the study include the heterogeneity of patients in the “no AI withdrawal” group and the fact that BMD was measured only once during the off-treatment interval. Furthermore, fracture data are not yet mature and thus not presented here.

In conclusion, this study is to our knowledge the first to quantify the long-term impact of AI withdrawal on BMD 5 years after treatment discontinuation in women with early breast cancer. Bone loss associated with AI therapy seems partially reversible after stopping treatment, particularly at the lumbar spine.

### Supplementary Information

Below is the link to the electronic supplementary material.

Supplementary file1 (DOCX 14 KB)

## Data Availability

The datasets generated during and/or analyzed during the current study are not publicly available due to the formulation in the consent form but are available from the corresponding author on reasonable request.
