# Supplementary material for: Long-term effects of aromatase inhibitor withdrawal on bone mineral density in early breast cancer patients: 10-year follow-up results of the BREX study
Source: Breast Cancer Res Treat. 2024 Apr 1;206(1):57–65. doi: 10.1007/s10549-024-07252-7 (PMC11182844; doi:10.1007/s10549-024-07252-7)
Supplement: Supplementary file 1 — Supplementary file1 (DOCX 14 KB) [file 10549_2024_7252_MOESM1_ESM.docx]

**Inclusion and exclusion criteria of the BREX study**

| **Inclusion criteria** |
| --- |
| Histologically proven invasive breast cancer T1-4 N0-3 M0 |
| Pre- or postmenopausal breast cancer patient treated with adjuvant chemotherapy or radiotherapy within 4 months, or patient who has started adjuvant endocrine therapy no less than 4 months earlier |
| Age between 35 and 68 years |
| Signed informed consent prior to beginning specific protocol procedures |
|  |
| **Exclusion criteria** |
| Male gender |
| Prior malignancy except basal cell carcinoma or in situ cervix carcinoma |
| Hematogenous metastases (M1) |
| No systemic adjuvant therapy |
| Postmenopausal women with antiestrogens as the only adjuvant therapy (with/without radiotherapy) |
| Pregnancy or recent lactation (<1 year) |
| Severe cardiac disease (NYHA class III or greater), myocardial infarction within 12 months, uncontrolled hypertension |
| Verified osteoporosis (proximal femur or lumbar spine T-score lower than -2.5, fracture without trauma) |
| Concomitant medications affecting calcium and bone metabolism such as bisphosphonates, calcitonin, parathormone (PTH), selective estrogen receptor modulators (SERMs), oral corticosteroids over 6 months, anticonvulsants (phenytoin or carbamazepine), prolonged heparin therapy |
| Other diseases affecting calcium and bone metabolism such as hyperthyroidism, newly diagnosed hypothyroidism, primary hyperparathyroidism, renal failure, chronic hepatic diseases, organ transplant |
| Other serious illness or medical condition, which could be contradiction to exercise |
| Patient not capable of training (severe knee arthrosis, severe ligament, or cartilage injuries of lower extremities) |
| Residence more than one hour from the exercise center |
| Competitive athlete |
